# Clinical significance of high monocyte counts for the continuous treatment with nintedanib

**DOI:** 10.1186/s12890-023-02536-y

**Published:** 2023-07-03

**Authors:** Shingo Tsuneyoshi, Yoshiaki Zaizen, Masaki Tominaga, Goushi Matama, Shushi Umemoto, Shuuhei Ohno, Reiko Takaki, Ryo Yano, Kenta Murotani, Masaki Okamoto, Tomoaki Hoshino

**Affiliations:** 1 Division of Respirology, Neurology and Rheumatology, Department of Medicine, Kurume University School of Medicine, 67 Asahi-machi, Kurume, 830-0011 Fukuoka Japan; 2 Department of Pathology, Nagasaki University Graduate School of Biomedical Sciences, 1-7-1 Sakamoto, Nagasaki, 852-8501 Japan; 3 Biostatistics Center, Kurume University, 67 Asahi-machi, Kurume, 830-0011 Japan; 4 Department of Respirology and Clinical Research Center, National Hospital Organization Kyushu Medical Center, 1-8-1 Jigyouhama, Chuo-ku, Fukuoka, 810-8563 Japan

**Keywords:** Nintedanib, Starting dosage, Monocyte count, Adverse events

## Abstract

**Background:**

Nintedanib is now widely used to treat interstitial lung disease (ILD). Adverse events, which occur in not a few patients, make it difficult to continue nintedanib treatment, but the risk factors for adverse events are not well understood.

**Methods:**

In this retrospective cohort study, we enrolled 111 patients with ILDs treated with nintedanib and investigated the factors involved in starting dosage reduction, withdrawal, or discontinuation within 12 months, even with appropriate symptomatic treatment. We also examined the efficacy of nintedanib in reducing the frequency of acute exacerbations and the prevention of pulmonary function reduction.

**Results:**

Patients with high monocyte counts (> 0.454 × 10^9^/L) had a significantly higher frequency of treatment failure, such as dosage reduction, withdrawal, or discontinuation. High monocyte count was as significant a risk factor as body surface area (BSA). Regarding efficacy, there was no difference in the frequency of acute exacerbations or the amount of decline in pulmonary function within 12 months between the normal (300 mg) and reduced (200 mg) starting dosage groups.

**Conclusion:**

Our study results indicate that patients with higher monocyte counts (> 0.454 × 109/L) should very careful about side effects with regard to nintedanib administration. Like BSA, a higher monocyte count is considered a risk factor for nintedanib treatment failure. There was no difference in FVC decline and frequency of acute exacerbations between the starting doseage of nintedanib, 300 mg and 200 mg. Considering the risk of withdrawal periods and discontinuation, a reduced starting dosage may be acceptable in the patients with higher monocyte counts or small body sizes.

## Introduction

Idiopathic pulmonary fibrosis (IPF) is a disease of unknown cause characterized by chronic and progressive fibrosis. IPF has a histopathologic UIP pattern and is rapidly progressive disease with a poor prognosis. The pathogenesis of IPF is not fully understood; however, recent reports have demonstrated the efficacy of the antifibrotic agents nintedanib and pirfenidone [1, 2]. Similarly, systemic sclerosis (SSc) is known to cause lung fibrosis. The clinical course of patients with sclerosis varies, and approximately 50% of them have interstitial lung disease (ILD) [3]. SSc-ILD has a histopathologic fibrotic nonspecific interstitial pneumonia(NSIP) pattern, and some cases show fibrosis progression, although more slowly than IPF. Recently, it has been reported that patients with ILDs (excluding IPF) may have a common form of progressive fibrosis [4] and the concept of progressive fibrosing ILD (PF-ILD) has been established. PF-ILDs include idiopathic nonspecific interstitial pneumonia, hypersensitivity pneumonitis, connective tissue disease-associated ILD, sarcoidosis, and so on. PF-ILD, as well as IPF, has been shown to have a poor prognosis [5]. In patients with any ILD that presents with chronic progressive fibrosis, prevention of acute exacerbations and decline in pulmonary function is important for disease control, prognosis, and maintenance of the patient’s quality of life. In addition, the relationship between high monocyte counts and poor prognosis in IPF has been reported in recent years and has attracted much attention [6, 7]. Nintedanib is a tyrosine kinase inhibitor that exerts inhibitory activity against the platelet-derived growth factor receptor, fibroblast growth factor receptor, and vascular endothelial growth factor receptor, thereby inhibiting fibrosis progression. Therefore, it is used as an antifibrotic agent in the treatment of many ILDs. Previous clinical trials have shown its efficacy in IPF, SS-associated ILD (SSc-ILD), and PF-ILD [1, 8, 9].

While nintedanib is used for the treatment of various ILDs, it causes adverse events in 71–82% of cases. The most frequent adverse events are gastrointestinal symptoms, liver disorders, and thrombocytopenia [1, 8–11]. Previous reports have shown that a low body surface area (BSA) is associated with nintedanib dosage reduction and discontinuation [12]. Moreover, a tendency for gastrointestinal symptoms and hepatotoxicity has been reported in smaller patients and in the normal starting dosage group,[8, 9, 13–16], but this has not been fully explored. In current real-world practice, the starting dosage is sometimes reduced. The trials of nintedanib included mainly Western patients, and the dosage may be too high for a small Japanese physique.

Symptomatic treatments for adverse events caused by nintedanib have also been established. Although drug withdrawal may occur due to adverse events, patients are at risk of acute exacerbations during the withdrawal period. We believe that continued and stable nintedanib administration is beneficial for ILD control. In this study, we aimed to investigate the tolerability and efficacy of nintedanib in real-world situations, where the nintedanib starting dosage was reduced and various symptomatic treatments were administered. We also analyzed factors that influence nintedanib administration by analyzing the patients’ backgrounds and found that a high monocyte count had a negative impact on the continuation of nintedanib administration.

## Materials and methods

### Study participants

This was a retrospective cohort study. We collected the clinical information of all patients who started nintedanib at Kurume University Hospital between September 2015 and September 2020. The IPF diagnosis was based on the official ATS/ERS/JRS/ALAT guidelines [17, 18]. PF-ILD was diagnosed based on the diagnostic criteria of the INBUILD study [19]. SSc-ILD had characteristic high-resolution computed tomography findings [20] and was diagnosed as SSc according to the 2013 classification criteria [21]. SSc-ILD that met the criteria for PF-ILD was considered SSc-ILD in this study. Clinical and laboratory data such as age, sex, smoking history, height, weight, body mass index (BMI), BSA, performance status, modified Medical Research Council Dyspnea Scale (mMRC) score, hematological findings, and pulmonary function test findings were retrieved from medical records.

### Evaluation of adverse events and efficacy of nintedanib

We examined the risk of adverse events that occur despite appropriate symptomatic treatment. We investigated the occurrence of adverse events within 12 months, requiring a change in the way nintedanib was administered, such as dosage reduction, withdrawal, or discontinuation. The appropriate symptomatic treatment was metoclopramide and domperidone for nausea, and probiotics, loperamide, and albumin tannate for diarrhea. Pulmonary function tests were performed before starting nintedanib and at 12 months after starting treatment. In the present study, in addition to patients who were started on nintedanib at 300 mg as usual, some patients were started on a reduced doseage of 200 mg. We compared the treatment starting dosage between the normal starting dosage group and the reduced starting dosage group. Efficacy was evaluated based on changes in pulmonary function tests at 12 months and the occurrence of acute exacerbations within 12 months.

### Statistical analysis

Unless mentioned otherwise, numerical values are presented as medians (25–75% interquartile ranges). The statistical significance of differences between the two study groups was analyzed using Wilcoxon’s rank-sum test or Fisher’s exact test. Multivariate analysis was performed using logistic regression analysis with reference to univariate results and factors were extracted using forward-backward stepwise procedure. Receiver operating characteristic (ROC) curve cut-off values were determined using the Youden Index. Statistical significance was defined as P < 0.05. All statistical analyses were performed using JMP Pro version 16.0 (SAS Institute, Cary, NC, USA).

## Results

### Patient characteristics

The current study enrolled 111 patients with ILD including IPF, SSc-ILD, or PF-ILD. Within 12 months, a defined adverse event occurred in 66 patients of which 47 reduced the dosage, 33 withdrew, and 21 discontinued the drug. Adverse events included diarrhea in 37 cases, nausea and anorexia in 20 cases, hepatotoxicity in 21 cases, and gastrointestinal hemorrhage in 2 cases.

The backgrounds of all patients (80 men and 31 women; median age 70 years [66–75]; 76 smokers) and of patients by nintedanib induction dosage are shown in Table 1. The diagnoses were IPF in 76 patients, SSc-ILD in 17 patients, and PF-ILD in 18 patients. Patients with PF-ILD included six cases of fibrotic hypersensitivity pneumonitis, five cases of connective tissue disease, one case of pleuroparenchymal fibroelastosis and six cases of unclassifiable ILD. The median BMI was 22.9 kg/m^2^ (20.6–25.2), and the median BSA was 1.62 m^2^ (1.51–1.72). Blood biochemical tests showed serum albumin of 3.87 g/dL (3.59–4.10), lactate dehydrogenase of 223 U/L (195–248), Krebs von den Lungen-6 (KL-6) of 943 U/mL (633–1463), and surfactant protein-D of 218 ng/mL (120–368). The median forced vital capacity (FVC), percent predicted FVC (%FVC), and percent predicted diffusing capacity of the lung for carbon monoxide (%DLco) were 2.18 L (1.61–2.86), 70.4% (57.1–87.4), and 56.3% (44.8–73.7), respectively. Due to dyspnea and severe cough, 2 and 33 patients were unable to perform FVC measurements at start and at 12 months, respectively. Due to severe cough, hypoxemia, and reduced FVC, 19 and 59 patients could not undergo DLco measurements at start and at 12 months, respectively. No one had a history of obvious drug-induced lung injury or severe drug allergy.

**Table 1:** Patient background in all patients and by nintedanib starting doseage

	Total	300 mg	200 mg	P-value
	(n = 111)	(n = 83)	(n = 28)	
Age (years)	70 (66–75)	69 (66–74)	73 (66–78)	0.1014
Male, n (%)	80 (72.1)	69 (83.1)	11 (39.3)	**< 0.0001**
IPF/PF-ILD/SSc-ILD	59/35/17	44/28/11	15/7/6	0.4966
Smoking (pack-years)	40 (20.0–57.6)	40 (22.0–64.0)	20 (6.3–47.5)	**0.0330**
BMI (kg/m^2^)	22.9 (20.6–25.2)	23.2 (21.1–25.2)	21.7 (19.9–24.5)	0.0534
BSA (m^2^)	1.62 (1.51–1.72)	1.64 (1.55–1.74)	1.48 (1.42–1.64)	**0.0002**
Albumin (g/dL)	3.87 (3.59–4.10)	3.90 (3.60–4.10)	3.81 (3.50–4.18)	1.0000
C-reactive protein (mg/dL)	0.28 (0.10–0.59)	0.24 (0.10–0.67)	0.29 (0.13–0.52)	0.8532
KL-6 (U/mL)	943 (633–1463)	953 (641–1334)	893 (617–1742)	0.8232
White blood cell count (x10^9^/L)	7.400 (6.000–9.700)	7.400 (5.900–9.200)	7.300 (6.050–10.712)	0.4530
Monocyte count (x10^9^/L)	0.451 (0.352–0.600)	0.466 (0.350–0.600)	0.409 (0.365–0.585)	0.7652
mMRC score*	1.62 ± 1.05	1.47 ± 0.11	2.07 ± 0.19	**0.0099**
FVC (L)	2.18 (1.61–2.86)	2.34 (1.78–2.92)	1.72 (1.38–2.52)	**0.0068**
%FVC (%)	70.4 (57.1–87.4)	71.0 (59.7–87.4)	67.8 (55.8–88.2)	0.4538
%DLco (%)	56.3 (44.8–73.7)	56.4 (46.0–72.5)	56.0 (44.1–77.5)	0.9647
FVC decline per year (mL)	−125 (− 323–+20)	−150 (− 330–−20)	−20 (− 290–+100)	0.1361
FVC decline per year (%)	−5.2 (− 15.2–+0.7)	−5.5 (− 14.9–−0.7)	−0.9 (− 17.0–+4.6)	0.3311
%DLco decline per year (%)	−7.9 (− 13.0–−2.7)	−7.8 (− 11.8–−2.6)	−9.7 (− 18.1–−3.7)	0.3846
Acute Ex, n (%)	11 (9.9)	10 (12.1)	1 (3.6)	0.2824
Adverse events with medication change, n (%)	66 (59.5)	55 (66.3)	11 (39.3)	0.0150

*The mMRC scores are presented as mean values ± standard deviations

IPF, Idiopathic pulmonary fibrosis; PF-ILD, progressive fibrosing interstitial lung disease; SSc-ILD, systemic sclerosis with interstitial lung disease, %DLco, percent predicted diffusing capacity of the lung for carbon monoxide; %FVC, percent predicted FVC; Acute Ex, acute exacerbation; BMI, body mass index; BSA, body surface area; FVC, forced vital capacity; KL-6, Krebs von den Lungen-6; mMRC, modified Medical Research Council Dyspnea Scale

### Comparison of normal and reduced dosage starting groups

The reduced and normal starting dosage groups comprised 28 and 83 patients, respectively. The reduced starting dosage group had significantly more female participants (60.7% vs. 16.9%, P < 0.0001), fewer cigarette pack-years (20 pack-years vs. 40 pack-years, P = 0.0330), lower BSA values (1.48 m^2^ vs. 1.64 m^2^, P = 0.0002), higher mMRC scores (1.47 vs. 2.07, P = 0.0099), and significantly fewer changes in the dosage of nintedanib administration (39.3% vs. 66.3%, P = 0.0150) than the normal starting dosage group. However, no statistically significant differences were found in the multivariate analysis.

**Fig. 1:**
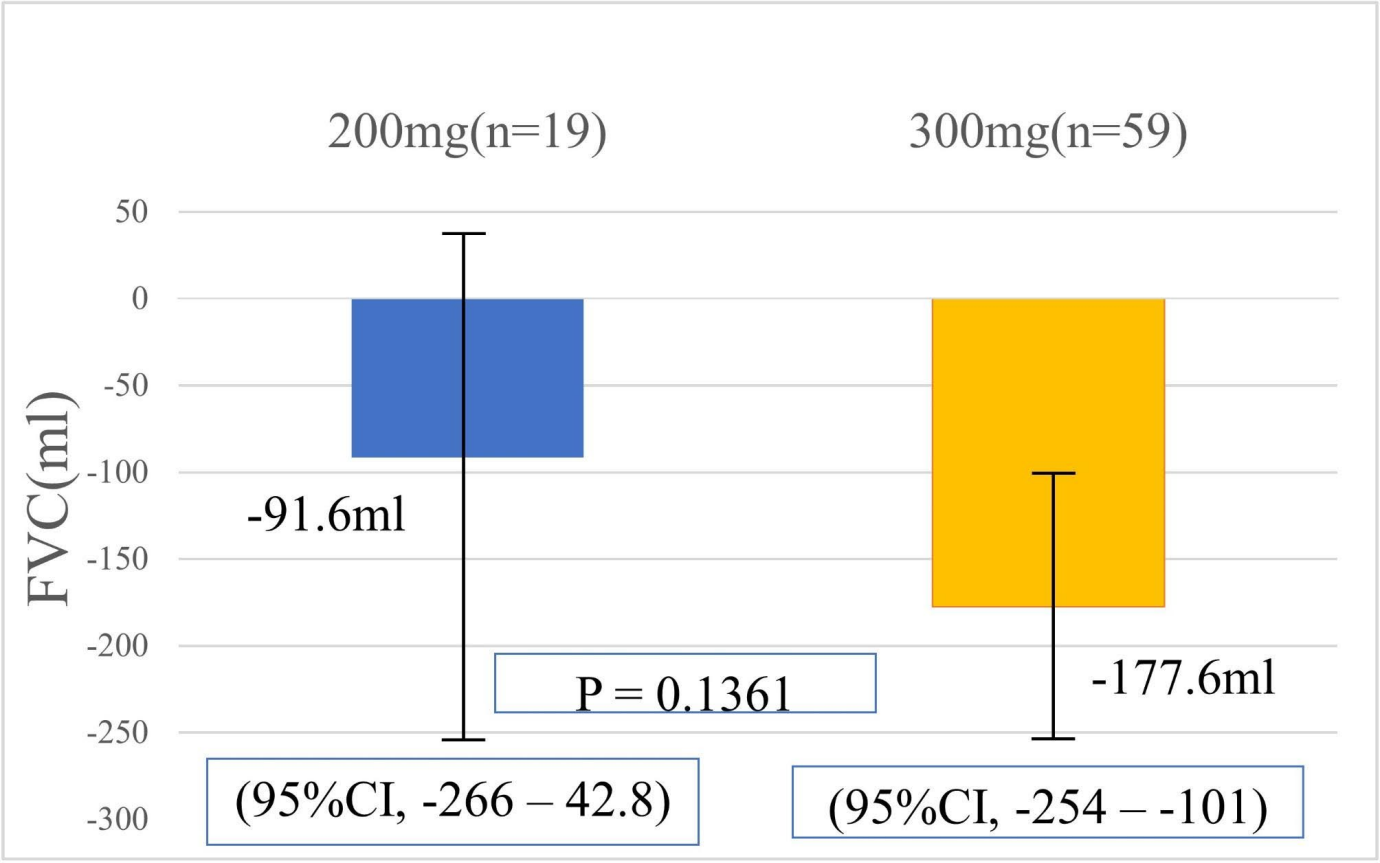
Decline in forced vital capacity (FVC) within one year by starting dosage. The average annual FVC reduction by starting dosage is shown. The reduced starting dosage group had an FVC reduction of − 91.6 mL (− 266–+42.8), and the normal starting dosage group had a reduction of − 177.6 mL (− 254–+101). No significant difference was found (P = 0.1361)

The median FVC decline per year was − 20 mL (− 290–+100) in the reduced starting dosage group and − 150 mL (− 330–−20) in the normal starting dosage group. The average FVC decline per year was − 91.6 mL (− 266–+42.8) in the reduced starting dosage group and − 177.6 mL (− 254–+101; Fig. 1) in the normal starting dosage group, with no significant between-group difference (P = 0.1361). The reduction in %DLco per year was − 9.7% (− 18.1–−3.7) in the reduced starting dosage group and − 7.8% (− 11.8–−2.6) in the normal starting dosage group, which was not significantly different between the two groups (P = 0.3846).

### Adverse events requiring changes in dosage regimen within 12 months

We performed a comparison between the group of 66 patients who experienced adverse events with nintedanib and the rest of the patients (Table 2). Adverse events occurred significant higher in patients with high BMI (23.2 kg/m^2^ vs. 22.1 kg/m^2^, P = 0.0332), high BSA (1.63 m^2^ vs. 1.56 m^2^, P = 0.0471), high monocyte count (0.454 × 10^9^/L vs. 0.410 × 10^9^/L, P = 0.0178), and in the normal starting dosage group (83.3% vs. 62.2%, P = 0.0150). There were no significant differences in age, serum albumin level, and liver or renal function.

**Table 2:** Adverse events requiring changes in dosage regimen, such as dosage reduction, withdrawal, or discontinuation within one year

	Total	With Adverse Event	Without Adverse Event	P-value
	(n = 111)	(n = 66)	(n = 45)	
Age (years)	70 (66–75)	70 (66–75.3)	71 (65–75)	0.7499
Male, n (%)	80 (72.1)	50 (75.8)	30 (66.7)	0.3890
IPF/PF-ILD/SSc-ILD	59/35/17	38/17/11	21/18/6	0.3120
Dosage reduction	47	47	0	
Dosage withdrawal	33	33	0	
Dosage discontinuation	21	21	0	
Smoking (pack-years)	40 (20–57.6)	40 (20–60)	40 (19.3–56.8)	0.9446
BMI (kg/m^2^)	22.9 (20.6–25.2)	23.2 (21.3–25.7)	22.1 (19.7–24.5)	**0.0332**
BSA (m^2^)	1.62 (1.51–1.72)	1.63 (1.56–1.73)	1.56 (1.46–1.69)	**0.0471**
Albumin	3.87 (3.59–4.10)	3.83 (3.52–4.10)	3.90 (3.61–4.11)	0.2898
C-reactive protein (mg/dL)	0.28 (0.10–0.59)	0.28 (0.10–0.77)	0.28 (0.08–0.50)	0.2382
KL-6 (U/mL)	943 (633–1463)	922 (626–1342)	993 (645–1694)	0.3663
White blood cell count (x10^9^/L)	7.400 (6.000–9.700)	7.550 (6.375–9.725)	6.800 (5.550–9.725)	0.2170
Monocyte count (x10^9^/L)	0.451 (0.352–0.600)	0.492 (0.385–0.630)	0.410 (0.397–0.557)	**0.0178**
mMRC score*	1.62 ± 1.05	1.58 ± 0.16	1.65 ± 0.13	0.8867
FVC	2.18 (1.61–2.86)	2.18 (1.69–2.89)	2.25 (1.54–2.85)	0.8073
%FVC	70.4 (57.1–87.4)	70.4 (55.4–87.6)	70.4 (59.3–83.5)	0.7645
%DLco	56.3 (44.8–73.7)	55.8 (46.6–71.4)	61.8 (44.4–76.9)	0.3962
Starting dosage 300 mg (%)	83 (74.8)	55 (83.3)	28 (62.2)	**0.0150**

*The mMRC scores are presented as mean values ± standard deviations

IPF, Idiopathic pulmonary fibrosis; PF-ILD, progressive fibrosing interstitial lung disease; SSc-ILD, systemic sclerosis with interstitial lung disease, %DLco, percent predicted diffusing capacity of the lung for carbon monoxide; %FVC, percent predicted FVC; AE, adverse events; BMI, body mass index; BSA, body surface area; FVC, forced vital capacity; KL-6, Krebs von den Lungen-6; mMRC, modified Medical Research Council Dyspnea Scale

The results of the multivariate analysis are presented in Table 3. The univariate analysis using logistic regression analysis showed significant differences in high BMI (23.2 kg/m^2^ vs. 22.1 kg/m^2^, P = 0.0258), high C-reactive protein (CRP) (0.28 mg/dL vs. 0.28 mg/dL, P = 0.313), high Monocyte count (0.454 × 10^9^/L vs. 0.410 × 10^9^/L, P = 0.0131), and in the normal starting dosage group (83.3% vs. 62.2%, P = 0.0125). Multivariate analysis showed higher odds ratios of adverse events with nintedanib in the high monocyte count. In contrast, BMI, CRP and starting dosage were not correlated with adverse events during nintedanib treatment in the multivariate analysis.

**Table 3:** Logistic regression analysis with forward-backward procedure for adverse event

	Total		With Adverse Event		Without Adverse Event		P-value(univariate)	Odds 95% CI	P-value(multiple)
	(n = 111)		(n = 66)		(n = 45)				
Age (years)	70 (66–75)		70 (66–75.3)		71 (65–75)		0.4957		
Male, n (%)	80 (72.1)		50 (75.8)		30 (66.7)		0.2968		
Smoking (pack-years)	40 (20–57.6)		40 (20–60)		40 (19.3–56.8)		0.6358		
BMI (kg/m^2^)	22.9 (20.6–25.2)		23.2 (21.3–25.7)		22.1 (19.7–24.5)		**0.0258**		N/A
BSA (m^2^)	1.62 (1.51–1.72)		1.63 (1.56–1.73)		1.56 (1.46–1.69)		0.0975		
Albumin	3.87 (3.59–4.10)		3.83 (3.52–4.10)		3.90 (3.61–4.11)		0.1893		
C-reactive protein (mg/dL)	0.28 (0.10–0.59)		0.28 (0.10–0.77)		0.28 (0.08–0.50)		**0.0313**		N/A
KL-6 (U/mL)	943 (633–1463)		922 (626–1342)		993 (645–1694)		0.4863		
White blood cell count (x10^9^/L)	7.400 (6.000–9.700)		7.550 (6.375–9.725)		6.800 (5.550–9.725)		0.5316		
Monocyte count (x10^9^/L)	0.451 (0.352–0.600)		0.492 (0.385–0.630)		0.410 (0.397–0.557)		**0.0131**	1.002 (1.005–1.005)	**0.0131**
mMRC score*	1.62 ± 1.05		1.58 ± 0.16		1.65 ± 0.13		0.7157		
FVC	2.18 (1.61–2.86)		2.18 (1.69–2.89)		2.25 (1.54–2.85)		0.8472		
%FVC	70.4 (57.1–87.4)		70.4 (55.4–87.6)		70.4 (59.3–83.5)		0.5954		
%DLco	56.3 (44.8–73.7)		55.8 (46.6–71.4)		61.8 (44.4–76.9)		0.2675		
Starting dosage 300 mg (%)	83 (74.8)		55 (83.3)		28 (62.2)		**0.0125**		N/A
Acute exacerbation		11(9.9%)		5(7.6%)		6(13.3%)	**0.3237**		

*The mMRC scores are presented as mean values ± standard deviations

%DLco, percent predicted diffusing capacity of the lung for carbon monoxide; %FVC, percent predicted FVC; AE, adverse events; BMI, body mass index; BSA, body surface area; FVC, forced vital capacity; KL-6, Krebs von den Lungen-6; mMRC, modified Medical Research Council Dyspnea Scale

We conducted subset analysis in CTD-ILD, but there was no significant difference in the incidence of adverse events with high monocyte counts in CTD-ILD.

### Monocyte count cut-off values according to ROC curves

**Fig. 2:**
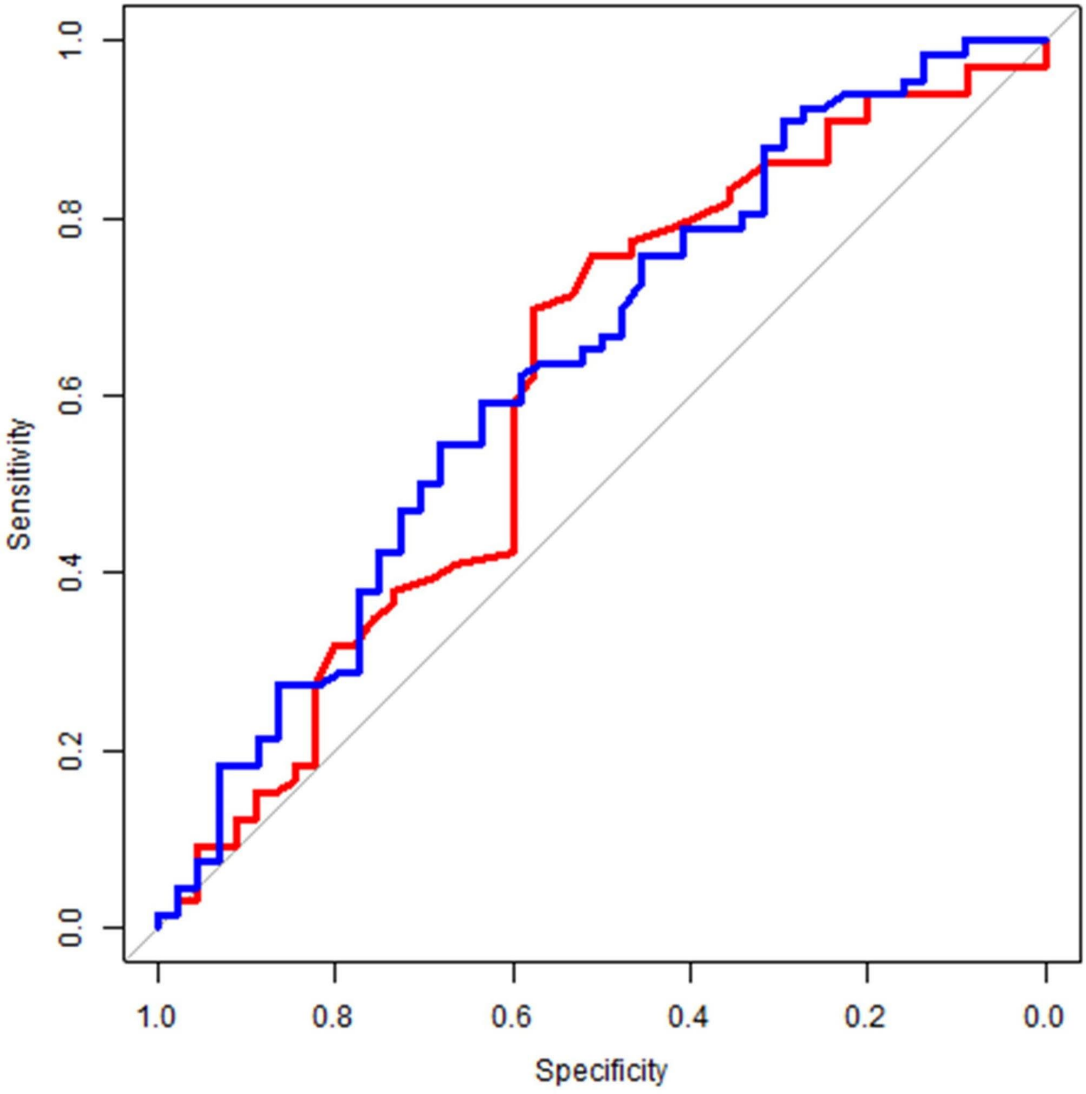
Receiver operating characteristic (ROC) curve analysis to determine the cut-off values for monocyte count and body surface area (BSA). The blue and red lines show the monocyte count and BSA, respectively. The area under the curve (AUC) for monocyte count was 0.63 (0.53–0.74), with a sensitivity of 0.59 and a specificity of 0.64. The cut-off value was 0.454 × 109/L. The AUC for BSA was 0.61 (0.50–0.72), with a sensitivity of 0.70 and specificity of 0.58. The cut-off value was 1.59 m^2^. The AUC value of the monocyte count are almost equal to that of the BSA. The cut-off value was determined as the value at which the Youden index was greatest

The ROC curves for the determination of cut-off values for monocyte counts predicting the occurrence of adverse events with nintedanib are shown in Fig. 2. The area under the curve (AUC) for monocyte counts was 0.63 (95% confidence interval, 0.53–0.74). The cut-off value with maximum sensitivity and specificity was 0.454 × 10^9^/L (sensitivity, 0.59; specificity, 0.64). The AUC value for monocyte count was almost comparable with that for BSA, which has been reported to be associated with the occurrence of nintedanib-associated adverse events.

### Acute exacerbation within 12 months

We also analyzed acute exacerbations while on medication within 12 months of starting nintedanib administration (Table 4). Eleven patients presented with acute exacerbations within 12 months. Male sex (100% vs. 69%, P = 0.0324), low serum albumin concentrations (3.30 g/dL vs. 3.90 g/dL, P = 0.0030), and high mMRC scores (2.63 vs. 1.51, P = 0.0009) were significantly more common in patients who developed an acute exacerbation. The gender, age, and lung physiology (GAP) stage (3.36 vs. 2.53, P = 0.0052) also showed a significantly increased risk of acute exacerbation. Moreover, the present study showed a significant difference in low pulmonary function, defined as %FVC ≤ 50% or %DLco ≤ 40% (55.6% vs. 22.0%, P = 0.0400). However, no statistical significance was found in the multivariate analysis.

**Table 4:** Acute exacerbation while on nintedanib medication within one year

	Total	With Acute Ex	Without Acute Ex	P-value
	(n = 111)	(n = 11)	(n = 100)	
Age (years)	69 (66–74)	70 (66–75.3)	70 (66–75.8)	0.8279
Male, n (%)	80 (72.1)	11 (100)	69 (69)	0.0324
IPF/PF-ILD/SSc-ILD	59/35/17	7/4/0	52/31/17	0.3828
Smoking (pack-years)	40 (18.5–82.8)	40 (20–60)	40 (20–58.5)	0.7599
BMI (kg/m^2^)	22.9 (20.6–25.2)	23.4 (20.3–24.8)	22.7 (20.6–25.3)	0.9214
BSA (m^2^)	1.62 (1.51–1.72)	1.68 (1.60–1.72)	1.61 (1.50–1.72)	0.1582
Albumin	3.87 (3.59–4.10)	3.3 (3.27–3.76)	3.9 (3.7–4.1)	0.0030
C-reactive protein (mg/dL)	0.28 (0.10–0.59)	0.56 (0.16–0.89)	0.24 (0.10–0.56)	0.0986
KL-6 (U/mL)	943 (633–1463)	1379 (946–1919)	908 (624–1361)	0.0552
White blood cell count (x10^9^/L)	7.400 (6.000–9.700)	8.400 (6.800–11.200)	7.200 (5.900–9.475)	0.0924
Monocyte count (x10^9^/L)	0.451 (0.352–0.600)	0.581 (0.296–0.694)	0.449 (0.358–0.580)	0.7612
mMRC score*	1.62 ± 1.05	2.63 ± 0.30	1.51 ± 0.10	0.0009
FVC	2.18 (1.61–2.86)	2.31 (1.59–2.69)	2.18 (1.60–2.88)	0.9517
%FVC	70.4 (57.1–87.4)	70.2 (47.1–77.4)	70.5 (59.2–87.7)	0.3137
%DLco	56.3 (44.8–73.7)	45.6 (37–55.8)	57.3 (46.6–74.7)	0.0667
Low pulmonary function, n (%)	27 (24.5)	5 (55.6)	22 (22)	0.0400
GAP stage*	2.61 ± 0.96	3.36 ± 0.28	2.53 ± 0.09	0.0052
Starting dosage 300 mg (%)	83 (74.8)	10 (90.1)	73 (73.0)	0.3137

*The mMRC scores and GAP stages are presented as mean values ± standard deviations

IPF, Idiopathic pulmonary fibrosis; PF-ILD, progressive fibrosing interstitial lung disease; SSc-ILD, systemic sclerosis with interstitial lung disease, %DLco, percent predicted diffusing capacity of the lung for carbon monoxide; %FVC, percent predicted FVC; Acute Ex, acute exacerbation; BMI, body mass index; BSA, body surface area; FVC, forced vital capacity; GAP stage, gender, age, and lung physiology stage; KL-6, Krebs von den Lungen-6; mMRC, modified Medical Research Council Dyspnea Scale

Low pulmonary function was defined as %FVC ≤ 50% or %DLco ≤ 40%.

## Discussion

In the current study, we observed more adverse events requiring a change in dosage regimen within 12 months of nintedanib treatment start in patients with high initial monocyte counts. A monocyte count > 0.454 × 10^9^/L was suggested as a risk factor for adverse events in patients treated with nintedanib. Recently, an increased monocyte count has been reported to be associated with IPF progression, hospital admission, and the risk of death [6] and as a predictor of poor prognosis [7, 22]. However, there were no previous reports on monocyte counts and nintedanib-associated adverse events. This is the first report to show that increased monocyte counts may be involved in the development of adverse events associated with nintedanib. We suspect that the inability to administer nintedanib on schedule due to adverse events may be a risk for poor prognosis.

In previous reports, low BSA was associated with dosage reduction and discontinuation of nintedanib [12]. Our study also analyzed BSA (Fig. 1). The AUC for BSA was similar to that for a high initial monocyte count. Like BSA, a high monocyte count at the treatment start may be a marker for changes in nintedanib medication. Furthermore, measuring monocyte counts is simple, less labor-intensive, and cost-effective. Therefore, we consider that monocyte counts is one of the good marker for predicting adverse nintedanib events like BSA. But the mechanism underlying high monocyte counts and nintedanib-associated adverse events remains unknown. This will be the subject of future research.

It has also been reported that, in Japanese patients, starting nintedanib at a normal dosage is associated with more adverse events [13–16]. This may be because Japanese people are approximately 30% lighter in body weight than Westerners [23]. Reduced disease progression has been reported in patients with long-term nintedanib treatment [10]. It should not be overlooked that in previous clinical trials, adverse events leading to nintedanib discontinuation occurred in approximately 20% of patients [1, 8, 9]. In clinical practice, 37.5% of patients cannot or are unwilling to restart nintedanib after treatment discontinuation [13]. We consider that treatment without adverse events not only improves continuation rates but also contributes to patient adherence. Therefore, it is important to reduce the number of adverse events. Based on previous reports,[8, 9, 13–16] the number of patients starting treatment with a reduced dosage of 200 mg has increased recently.

In agreement with previous reports, the reduced starting dosage group in our study population had significantly more female patients and a lower BSA. A previous report suggested a starting dosage reduction with a BSA cut-off of 1.58 m^2^.^[9]^ Our findings suggest a BSA cut-off value of 1.59 m^2^ to reduce adverse events resulting in changes in medication. For smaller Japanese, a reduction in the nintedanib starting dosage could be a good option.

In the present results, there were no significant differences by starting dosage in adverse events that caused a change in dosage in the multivariate analysis. These results may suggest that there is little need to reduce the starting doseage. However, there is no significant difference in efficacy for FVC decline or frequency of acute exacerbations, and dosage reduction may be beneficial in patients at high risk for adverse events, with high monocyte counts or lower BSA patients reported in previous reports.

In the current study, the efficacy measures covered the incidence of acute exacerbations and a decline in pulmonary function volume during the 12 months of treatment. The incidence of acute exacerbations was not significantly difference between the normal starting dosage group and the reduced starting dosage group. A high monocyte count has been reported as a risk factor for acute ILD exacerbations [24]. However, our study did not find any significant difference. The incidence of acute exacerbation did not reach statistical significance; however, the rate of acute exacerbations was lower in the reduced starting dosage group (3.6% vs. 12.1%). This was expected due to the lack of statistical power caused by the small number of cases with acute exacerbations.

The reduction of pulmonary function within 12 months was not significantly different between the normal starting dosage group and the reduced starting dosage group. Our study included patients with a low BSA in the normal starting dosage group. Therefore, the normal starting dosage group may have had more withdrawals and discontinuations, which may have reduced the medication effect. These results were similar to those of a similar report that found no significant difference in FVC change 6 months after nintedanib introduction between the reduced starting dosage group and the normal starting dosage group [25].

The current study has some limitations. This study had a retrospective single-center design, and the small number of patients was a limitation of this study. In particular, few patients had acute exacerbations. A larger prospective study is needed to confirm the results of this study. Other limitation is that the study period was short, preventing the assessment of long-term safety and efficacy. In addition, this study included patients with different diseases, IPF, SSc-ILD, and PF-ILD. Furthermore, the diagnosis of adverse events is made only by the patient’s attending doctor.

## Conclusion

Our findings suggest that high initial monocyte counts is a risk factor for adverse events that require medication changes for nintedanib. BSA has been previously reported and considered useful in predicting adverse events, but this study showed that high monocyte counts are also useful in predicting the risk of adverse events. High monocyte counts are a poor prognostic factor in ILD and may be related to the tolerability of nintedanib when the counts are elevated above 0.454 × 10^9^/L. There were no statistically significant differences in efficacy between the two starting dosage groups with regard to FVC decline and frequency of acute exacerbations. However, previous reports have reported differences in the frequency of adverse events not limited to changes in dosing regimen. Therefore, in patients with high monocyte counts, reducing the starting doseage of nintedanib may facilitate long-term administration of nintedanib and improve prognosis.

## Data Availability

The datasets used and/or analyzed during the current study available from the corresponding author on reasonable request.

## Abbreviations

%DLco: Percent predicted diffusing capacity of the lung for carbon monoxide
%FVC: Percent predicted FVC
AUC: Area under the curve
BMI: Body mass index
BSA: Body surface area
FVC: Forced vital capacity
GAP: Gender, age, and lung physiology
ILD: Interstitial lung disease
IPF: Idiopathic pulmonary fibrosis
KL-6: Krebs von den Lungen-6
mMRC: Modified Medical Research Council Dyspnea Scale
PF-ILD: Progressive fibrosing interstitial lung disease
ROC: Receiver operating characteristic
SS: Systemic sclerosis
Ssc-ILD: Interstitial lung disease associated with systemic sclerosis

## References

[1] Richeldi L, du Bois RM, Raghu G, Azuma A, Brown KK, Costabel U, Cottin V, Flaherty KR, Hansell DM, Inoue Y (2014). Efficacy and safety of nintedanib in idiopathic pulmonary fibrosis. N Engl J Med.

[2] King TE, Bradford WZ, Castro-Bernardini S, Fagan EA, Glaspole I, Glassberg MK, Gorina E, Hopkins PM, Kardatzke D, Lancaster L (2014). A phase 3 trial of pirfenidone in patients with idiopathic pulmonary fibrosis. N Engl J Med.

[3] Walker UA, Tyndall A, Czirják L, Denton C, Farge-Bancel D, Kowal-Bielecka O, Müller-Ladner U, Bocelli-Tyndall C, Matucci-Cerinic M (2007). Clinical risk assessment of organ manifestations in systemic sclerosis: a report from the EULAR Scleroderma trials and Research group database. Ann Rheum Dis.

[4] Olson A, Hartmann N, Patnaik P, Wallace L, Schlenker-Herceg R, Nasser M, Richeldi L, Hoffmann-Vold AM, Cottin V (2021). Estimation of the prevalence of Progressive Fibrosing interstitial lung Diseases: systematic literature review and data from a Physician Survey. Adv Ther.

[5] Simpson T, Barratt SL, Beirne P, Chaudhuri N, Crawshaw A, Crowley LE, Fletcher S, Gibbons MA, Hallchurch P, Horgan L et al. The burden of progressive fibrotic interstitial lung disease across the UK. Eur Respir J 2021, 58(1). 10.1183/13993003.00221-2021 PMC8264777 33678609

[6] Kreuter M, Lee JS, Tzouvelekis A, Oldham JM, Molyneaux PL, Weycker D, Atwood M, Kirchgaessler KU, Maher TM (2021). Monocyte Count as a prognostic biomarker in patients with idiopathic pulmonary fibrosis. Am J Respir Crit Care Med.

[7] Scott MKD, Quinn K, Li Q, Carroll R, Warsinske H, Vallania F, Chen S, Carns MA, Aren K, Sun J (2019). Increased monocyte count as a cellular biomarker for poor outcomes in fibrotic diseases: a retrospective, multicentre cohort study. Lancet Respir Med.

[8] Flaherty KR, Wells AU, Cottin V, Devaraj A, Walsh SLF, Inoue Y, Richeldi L, Kolb M, Tetzlaff K, Stowasser S (2019). Nintedanib in Progressive Fibrosing interstitial lung Diseases. N Engl J Med.

[9] Distler O, Highland KB, Gahlemann M, Azuma A, Fischer A, Mayes MD, Raghu G, Sauter W, Girard M, Alves M (2019). Nintedanib for systemic Sclerosis-Associated interstitial lung disease. N Engl J Med.

[10] Crestani B, Huggins JT, Kaye M, Costabel U, Glaspole I, Ogura T, Song JW, Stansen W, Quaresma M, Stowasser S (2019). Long-term safety and tolerability of nintedanib in patients with idiopathic pulmonary fibrosis: results from the open-label extension study, INPULSIS-ON. Lancet Respir Med.

[11] Richeldi L, Costabel U, Selman M, Kim DS, Hansell DM, Nicholson AG, Brown KK, Flaherty KR, Noble PW, Raghu G (2011). Efficacy of a tyrosine kinase inhibitor in idiopathic pulmonary fibrosis. N Engl J Med.

[12] Toi Y, Kimura Y, Domeki Y, Kawana S, Aiba T, Ono H, Aso M, Tsurumi K, Suzuki K, Shimizu H (2019). Association of low body surface area with dose reduction and/or discontinuation of nintedanib in patients with idiopathic pulmonary fibrosis: a pilot study. Sarcoidosis Vasc Diffuse Lung Dis.

[13] Ikeda S, Sekine A, Baba T, Yamanaka Y, Sadoyama S, Yamakawa H, Oda T, Okuda R, Kitamura H, Okudela K (2017). Low body surface area predicts hepatotoxicity of nintedanib in patients with idiopathic pulmonary fibrosis. Sci Rep.

[14] Kato M, Sasaki S, Nakamura T, Kurokawa K, Yamada T, Ochi Y, Ihara H, Takahashi F, Takahashi K (2020). Author correction: gastrointestinal adverse effects of nintedanib and the associated risk factors in patients with idiopathic pulmonary fibrosis. Sci Rep.

[15] Hirasawa Y, Abe M, Terada J, Sakayori M, Suzuki K, Yoshioka K, Kawasaki T, Tsushima K, Tatsumi K (2020). Tolerability of nintedanib-related diarrhea in patients with idiopathic pulmonary fibrosis. Pulm Pharmacol Ther.

[16] Uchida Y, Ikeda S, Sekine A, Katano T, Tabata E, Oda T, Okuda R, Kitamura H, Baba T, Komatsu S (2021). Tolerability and safety of nintedanib in elderly patients with idiopathic pulmonary fibrosis. Respir Investig.

[17] Raghu G, Remy-Jardin M, Myers JL, Richeldi L, Ryerson CJ, Lederer DJ, Behr J, Cottin V, Danoff SK, Morell F (2018). Diagnosis of idiopathic pulmonary fibrosis. An Official ATS/ERS/JRS/ALAT Clinical Practice Guideline. Am J Respir Crit Care Med.

[18] Raghu G, Remy-Jardin M, Richeldi L, Thomson CC, Inoue Y, Johkoh T, Kreuter M, Lynch DA, Maher TM, Martinez FJ (2022). Idiopathic pulmonary fibrosis (an update) and progressive pulmonary fibrosis in adults: an Official ATS/ERS/JRS/ALAT Clinical Practice Guideline. Am J Respir Crit Care Med.

[19] Richeldi L, Kreuter M, Selman M, Crestani B, Kirsten AM, Wuyts WA, Xu Z, Bernois K, Stowasser S, Quaresma M (2018). Long-term treatment of patients with idiopathic pulmonary fibrosis with nintedanib: results from the TOMORROW trial and its open-label extension. Thorax.

[20] Desai SR, Veeraraghavan S, Hansell DM, Nikolakopolou A, Goh NS, Nicholson AG, Colby TV, Denton CP, Black CM, du Bois RM (2004). CT features of lung disease in patients with systemic sclerosis: comparison with idiopathic pulmonary fibrosis and nonspecific interstitial pneumonia. Radiology.

[21] van den Hoogen F, Khanna D, Fransen J, Johnson SR, Baron M, Tyndall A, Matucci-Cerinic M, Naden RP, Medsger TA, Carreira PE (2013). 2013 classification criteria for systemic sclerosis: an american college of rheumatology/European league against rheumatism collaborative initiative. Ann Rheum Dis.

[22] Karampitsakos T, Torrisi S, Antoniou K, Manali E, Korbila I, Papaioannou O, Sampsonas F, Katsaras M, Vasarmidi E, Papakosta D (2021). Increased monocyte count and red cell distribution width as prognostic biomarkers in patients with idiopathic pulmonary fibrosis. Respir Res.

[23] Noble PW, Albera C, Bradford WZ, Costabel U, Glassberg MK, Kardatzke D, King TE, Lancaster L, Sahn SA, Szwarcberg J (2011). Pirfenidone in patients with idiopathic pulmonary fibrosis (CAPACITY): two randomised trials. Lancet.

[24] Kawamura K, Ichikado K, Anan K, Yasuda Y, Sekido Y, Suga M, Ichiyasu H, Sakagami T (2020). Monocyte count and the risk for acute exacerbation of fibrosing interstitial lung disease: a retrospective cohort study. Chron Respir Dis.

[25] Ikeda S, Sekine A, Baba T, Katano T, Tabata E, Shintani R, Yamakawa H, Niwa T, Oda T, Okuda R (2019). Low starting-dosage of nintedanib for the reduction of early termination. Respir Investig.

