# Correction to: clinical significance of high monocyte counts for the continuous treatment with nintedanib

**DOI:** 10.1186/s12890-023-02573-7

**Published:** 2023-07-21

**Authors:** Shingo Tsuneyoshi, Yoshiaki Zaizen, Masaki Tominaga, Goushi Matama, Shushi Umemoto, Shuuhei Ohno, Reiko Takaki, Ryo Yano, Kenta Murotani, Masaki Okamoto, Tomoaki Hoshino

**Affiliations:** 1 Division of Respirology, Neurology and Rheumatology, Department of Medicine, Kurume University School of Medicine, 67 Asahi-machi, Kurume, Fukuoka, 830-0011 Japan; 2 Department of Pathology, Nagasaki University Graduate School of Biomedical Sciences, 1-7-1 Sakamoto, Nagasaki, 852-8501 Japan; 3 Biostatistics Center, Kurume University, 67 Asahi-machi, Kurume, 830-0011 Japan; 4 Department of Respirology and Clinical Research Center, National Hospital Organization Kyushu Medical Center, 1-8-1 Jigyouhama, Chuo-ku, Fukuoka, 810-8563 Japan

Following publication of the original article, it came to the authors’ attention that the captions of Figs.1 and 2 had been erroneously swapped. The figures have since been corrected in the published article. The authors thank you for reading this correction and apologize for any inconvenience caused.

